# Efgartigimod in generalized myasthenia gravis: A real‐life experience at a national reference center

**DOI:** 10.1111/ene.16189

**Published:** 2024-01-02

**Authors:** Rita Frangiamore, Elena Rinaldi, Fiammetta Vanoli, Francesca Andreetta, Emilio Ciusani, Silvia Bonanno, Lorenzo Maggi, Annamaria Gallone, Anna Colasuonno, Irene Tramacere, Marta Cheli, Alessandro Pinna, Renato Mantegazza, Carlo Antozzi

**Affiliations:** ^1^ Neuroimmunology and Neuromuscular Diseases Unit Fondazione IRCCS Istituto Neurologico C. Besta Milan Italy; ^2^ Department of Human Neurosciences Sapienza University of Rome Rome Italy; ^3^ Laboratory of Neurological Biochemistry and Neuropharmacology Fondazione IRCCS Istituto Neurologico C. Besta Milan Italy; ^4^ Department of Research and Clinical Development, Scientific Directorate Fondazione IRCCS Istituto Neurologico C. Besta Milan Italy; ^5^ argenx Italy Milan Italy; ^6^ Immunotherapy and Apheresis Departmental Unit Fondazione IRCCS Istituto Neurologico C. Besta Milan Italy

**Keywords:** acetylcholine receptor, efgartigimod, MuSK, myasthenia gravis, neonatal Fc receptor

## Abstract

**Background and purpose:**

Inhibition of the neonatal Fc receptor (FcRn) for IgG is a promising new therapeutic strategy for antibody‐mediated disorders. We report our real‐life experience with efgartigimod (EFG) in 19 patients with generalized myasthenia gravis (gMG) along a clinical follow‐up of 14 months.

**Methods:**

EFG was administered according to the GENERATIVE protocol (consisting of a Fixed period of two treatment cycles [given 1 month apart] of four infusions at weekly intervals, followed by a Flexible period of re‐cycling in case of worsening). Eight patients were positive for acetylcholine receptor antibody, four for muscle‐specific tyrosine kinase antibody, and two for lipoprotein‐related protein 4 antibody, and five were classified as triple negative. Efficacy of EFG was assessed by the Myasthenia Gravis Activities of Daily Living, Myasthenia Gravis Composite, and Quantitative Myasthenia Gravis scales.

**Results:**

Fifty‐three percent of patients needed three treatment cycles, 26% needed four, and 21% needed five along the 14‐month clinical follow‐up. Meaningful improvement was observed at the end of each cycle with the clinical scores adopted. EFG had a dramatic effect on disease course, as during the year before treatment eight of 19 patients (42%) were hospitalized, and 15 of 19 (79%) needed treatment with plasma exchange or immunoglobulins; three of 19 (16%) were admitted to the intensive care unit. During EFG, none of the patients was hospitalized and only one patient required plasma exchange and intravenous immunoglobulins. No major side effects or infusion‐related reactions occurred.

**Conclusions:**

We observed that EFG was safe and modified significantly the course of the disease along a 14‐month follow‐up. Our experience strengthens the role of FcRn inhibition as an effective new tool for long‐term treatment of gMG.

## INTRODUCTION

Myasthenia gravis (MG) is a rare autoimmune disorder caused by specific antibodies against antigenic determinants of the neuromuscular junction, the main being nicotinic acetylcholine receptor (AChR), less frequently muscle‐specific tyrosine kinase (MuSK) or lipoprotein‐related protein 4 (LRP4) [1, 2]. Pathogenicity of specific antibodies has been demonstrated by robust experimental data, as well as the clinical observation of improvement correlated in time with immunomodulatory treatments such as plasma exchange (Plex) or intravenous immunoglobulins (IVIG) [3, 4]. Current pharmacological treatment of MG, including anticholinesterase inhibitors, corticosteroids, and immunosuppressive drugs, improves a considerable proportion of patients, but experience over several decades has unequivocally highlighted the burden of the disease, treatment side effects (particularly from corticosteroids), and urgent need for effective, targeted, and well‐tolerated therapies [5, 6]. Moreover, approximately 10% of MG patients can be refractory to available treatments, and comorbidities may hinder prolonged corticosteroid administration, particularly in elderly patients [7, 8, 9].

The pathogenesis of MG, a prototypic IgG antibody‐mediated disease, offers the extraordinary opportunity to investigate new therapies, and several randomized trials have paved the way to the era of targeted antibody therapy for MG [10].

Three main groups of biologics have been developed for clinical use, targeting the neonatal Fc receptor (FcRn), the complement cascade, and B cells [11]. The FcRn is a multifunctional Fc‐gamma receptor [12, 13] that during fetal life promotes the transplacental transfer of maternal antibodies to the fetus. In the adult, the FcRn binds circulating IgG and albumin that are rescued from intracellular lysosomal degradation and then released into the extracellular space. Due to this process, the half‐life of circulating IgGs (and hence pathogenic IgG) is increased several fold over that of IgA and IgM, which are not recycled by the FcRn. Therefore, inhibition of the FcRn increases IgG catabolism, as well as that of autoantibodies, providing a novel therapeutic opportunity for IgG‐mediated autoimmune disorders, including MG [14].

The FcRn antagonist efgartigimod (EFG) is a humanized IgG1 Fc fragment, engineered by ABDEG (AntiBody that enhances IgG DEGradation) technology, with increased affinity for the FcRn at neutral and acidic pH. The rapid efficacy and safety of EFG in MG was first reported in a phase 2 randomized study, and confirmed by the phase 3 ADAPT trial (NCT02965573, NCT03669588) [15, 16]. The results obtained by the ADAPT study led to approval of EFG in the USA in December 2021, and in Japan and Europe in 2022 [17].

The Expanded Early Access Program (EAP) to EFG for patients affected with generalized MG (gMG; GENERATIVE [GENERalized myAsThenia graVis Efgartgimod]) was launched in Italy in September 2021. We report our real‐world experience with EFG in 19 patients with gMG along a clinical follow‐up of 14 months.

## METHODS

### Treatment protocol

The EAP (version 1.0) was proposed with the rationale of providing access to EFG to patients with gMG before regulatory approval (protocol GENERATIVE version 1.0, 10 September 2021).

#### Inclusion criteria

The following inclusion criteria had to be met:

- At least 18 years of age at the time of signing the informed consent;
- Diagnosis of MG, with generalized muscle weakness supported by one of the following criteria: (1) AChR antibody seropositive or seronegative, (2) history of abnormal neuromuscular transmission demonstrated by single‐fiber electromyography or repetitive nerve stimulation, (3) history of positive edrophonium chloride test, or (4) demonstrated improvement in MG signs upon treatment with oral anticholinesterase inhibitors as assessed by the treating physician;
- Myasthenia Gravis Foundation of America (MGFA) classification II, III, IVa, or IVb [18];
- Myasthenia Gravis Activity of Daily Living (MG‐ADL) total score of ≥5 points, with >50% of the total score attributed to nonocular symptoms, or at least two nonocular items had to be scored at ≥2;
- Patient has been vaccinated against COVID‐19, or has had a negative COVID‐19 test result in the 2 weeks before enrollment; and
- Total IgG ≥ 6 g/L within 1 month of screening.

#### Treatment schedule

EFG was administered intravenously at a dose of 10 mg/kg as a 1‐h infusion in cycles of four weekly infusions. The initial Fixed period included two 3‐week treatment periods, each one followed by a 4‐week fixed intertreatment period. Afterward, according to the EAP protocol, patients were eligible to enter the Flexible cyclical period, during which EFG was given again as four weekly infusions at the discretion of the treating physician in the case of worsening of MG‐ADL and Quantitative Myasthenia Gravis (QMG) scores, as reported below.

EFG was provided by argenx according to the EAP in Italy.

### Clinical assessment of MG

Clinical assessment was performed according to the following clinical scales specific for MG: (i) MG‐ADL [19], (ii) Myasthenia Gravis Composite (MGC) [20], and (iii) QMG [21].

The MG‐ADL and MGC scores were administered at each infusion of every treatment cycle, and at each follow‐up visit performed 30 days after the end of each cycle of four infusions.

The QMG score was recorded before the first and fourth infusion of each cycle, and at each follow‐up visit performed 30 days after the end of each cycle.

MG‐ADL improvement was defined as at least a 2‐point reduction of the score; MGC and QMG improvement was defined as at least 3‐point reduction in the total QMG and MGC scores [21, 22, 23]. Increase of at least 2 points for the MG‐ADL score and 3 points for QMG and MGC scores was considered clinical criteria for prescription of retreatment with EFG along the clinical follow‐up.

Changes in MG‐ADL, MGC, and QMG scores were calculated comparing each time point to the score recorded before the first infusion of each treatment cycle.

Quality of life was evaluated by means of the revised 15‐item Myasthenia Gravis Quality of Life (MG‐QoL‐15r) questionnaire [24] at each infusion and follow‐up visit performed 30 days after the end of each cycle.

Ongoing treatment with cholinesterase inhibitors and immunosuppressive drugs remained unchanged during the study. The dose of corticosteroids was stable along the Fixed period, and tapered during the Flexible period at physician discretion.

### Laboratory investigations

Blood samples were collected for anti‐AChR antibody and total IgG assay before the first and fourth visit of each treatment cycle, and at follow‐up visits performed 30 days after the second, third, fourth, and fifth cycles. Anti‐AChR and MuSK antibodies were assayed by radioimmunoprecipitation (RSR). LRP4 antibody was assayed by homemade cell‐based assay [25]. IgG were measured by the Abbot Architect instrument. Patients with negative assays for Anti‐AChR, MuSK, and LRP4 antibodies were defined as triple‐negative MG.

### Informed consent

All patients signed informed consent before enrollment in the GENERATIVE program. Enrollment of each patient was possible after the EAP approval by the ethical committee of the Fondazione IRCCS Istituto Neurologico C. Besta of Milan, subsequently authorized by the Italian Medicines Agency.

### Statistical analysis

Descriptive statistics were provided in terms of absolute numbers and percentages for categorical data and mean with SE for continuous data. Changes in continuous outcomes were analyzed through a mixed effect model for repeated measures followed by Dunnett multiple comparisons tests. Probability values < 0.05 were considered statistically significant, and all tests were two‐sided. Prism version 8.4.2 for Windows (GraphPad Software, www.graphpad.comsoftware) was used for the statistical analysis.

## RESULTS

Nineteen patients affected by gMG were included in the EAP program from November 2021 and followed up to January 2023 for 14 months at the IRCCS Fondazione IRCCS Istituto Neurologico C. Besta of Milan.

The main demographic and baseline clinical characteristics of the patients are summarized in Table 1.

**TABLE 1:** Baseline characteristics of AChR, MuSK, and LRP4 Ab‐positive and triple‐negative gMG patients.

Characteristic	AChR‐Ab MG, *n* = 8 (42.1%)	MuSK‐Ab MG, *n* = 4 (21%)	LRP4‐Ab MG, *n* = 2 (10.6%)	Triple‐negative MG, *n* = 5 (26.3%)
Age, years (±SD)	52.3 (±16)	57.2 (±9.5)	45.5 (±2.5)	54.4 (±9.4)
Sex, *n* (%)				
Male	3 (37.5%)	1 (25%)	2 (100%)	0
Female	5 (62.5%)	3 (75%)	0	5 (100%)
MG duration, years (±SD)	10.8 (±7.7)	4.7 (±5.1)	8.5 (±7.5)	12 (±8.7)
Age at diagnosis, years (±SD)	41.3 (±15.5)	52.5 (±12)	37 (±10)	42.2 (±6.3)
MGFA at screening, *n* (%)				
IIa	0	1 (25%)	0	1 (20%)
IIb	2 (25%)	0	0	0
IIIa	3 (37.5%)	0	1 (50%)	2 (40%)
IIIb	2 (25%)	5 (50%)	1 (50%)	2 (40%)
IVa	0	0	0	0
IVb	1 (12.5%)	1 (25%)	0	0
Baseline scores (±SD)				
QMG	10.2 (±2.7)	11.7 (±7.2)	11.5 (±0.5)	13.6 (±4.5)
MG‐ADL	7.3 (±2.5)	8 (±4.6)	8.2 (±1.5)	9.6 (±1.7)
MGC	11.8 (±5.3)	12.7 (±7.2)	8.5 (±4.5)	16 (±4.8)
Ongoing therapies				
Anti‐AChE, *n* (%)	6 (75%)	0	0	3 (60%)
IS alone, *n* (%)	0	1 (25%)	0	0
Prednisone, *n* (%)	3 (37.5%)	2 (50%)	0	1 (20%)
Prednisone + IS, *n* (%)	5 (62.5%)	1 (25%)	0	3 (60%)
Prednisone, mg (±SD)	34.8 (±12.4)	47.5 (±23.5)	0	33.7 (±12.9)
Azathioprine, mg	150	150	0	0
Mycophenolate, mg	2000	0	0	0
Methotrexate, mg	0	0	0	12.5
Thymectomy, *n* (%)	3 (37.5%)	0	1 (50%)	3 (60%)
Thymoma, *n* (%)	1 (12.5%)	0	0	1 (20%)

Abbreviations: AChR, acetylcholine receptor; Anti‐AChE, acetylcholinesterase inhibitors; gMG, generalized MG; IS, immunosuppressive drugs; LRP4, lipoprotein‐related protein 4; MG, myasthenia gravis; MG‐ADL, Myasthenia Gravis Activity of Daily Living; MGC, Myasthenia Gravis Composite; MGFA, Myasthenia Gravis Foundation of America; MuSK, muscle‐specific tyrosine kinase; QMG, Quantitative Myasthenia Gravis.

The majority of patients were classified as MGFA classes IIb to IIIb; the severity of the disease at baseline was comparable among the four groups, with a trend for slightly more severe disease observed in seronegative patients. Ongoing treatments included a variable combination of cholinesterase inhibitors, corticosteroids, and immunosuppressive drugs in all patients except for the two LRP4‐MG subjects who received immunosuppression before starting EFG but from whom it was withdrawn due to side effects (Table 1).

**FIGURE 1:**
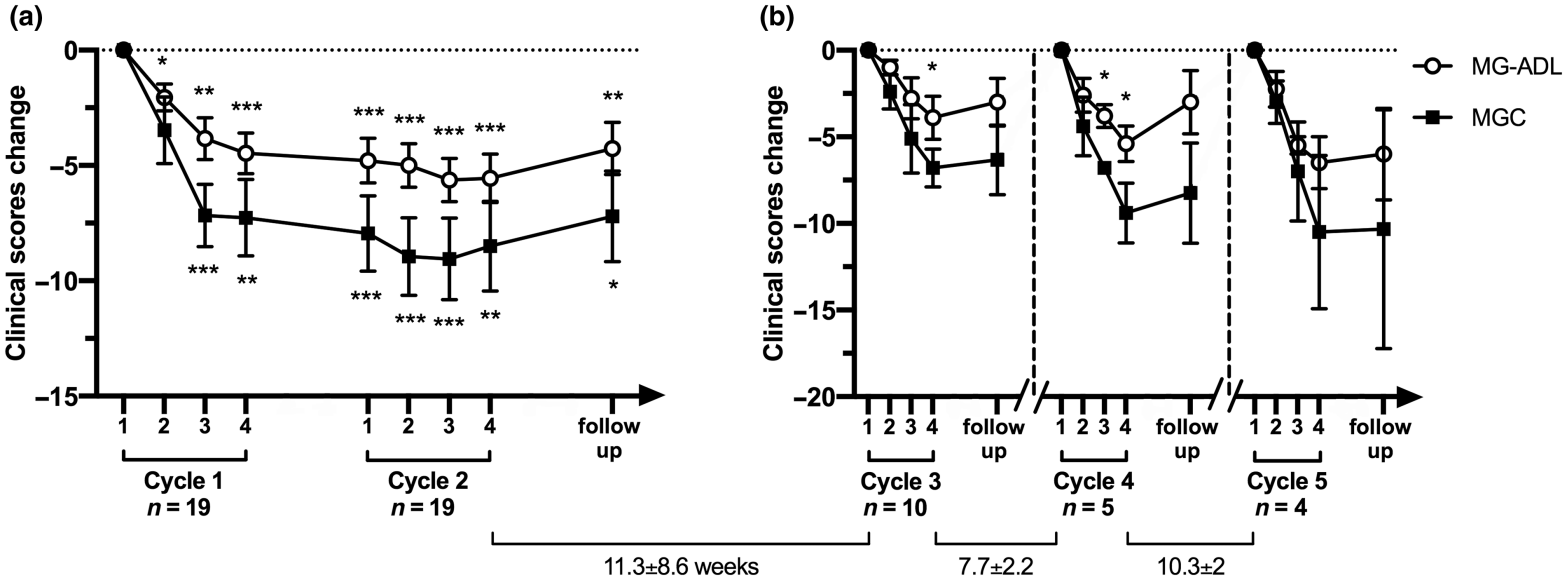
Myasthenia Gravis Activity of Daily Living (MG‐ADL) and Myasthenia Gravis Composite (MGC) mean score changes recorded during the Fixed period (a) and the Flexible Period (b) in generalized myasthenia gravis patients treated with efgartigimod (EFG). Changes in MG‐ADL and MGC (expressed as reduction of the score values, mean ± SEM) were calculated comparing each time point to the score recorded at the first infusion of each cycle. The time interval between EFG cycles during the Flexible period are reported as weeks (mean ± SD). *p < 0.05, **p < 0.01 ***p < 0.001.

We first evaluated the degree of clinical improvement in the whole group of MG patients during the Fixed treatment period (cycle 1 and cycle 2). Improvement in the MG‐ADL score was observed as early as the first treatment cycle and maintained by the second one for all patients; the observation was confirmed by MGC scores (Figure 1a). Clinical improvement was still recorded at the follow‐up visit performed 1 month after the end of the Fixed treatment period (Figure 1a).

Raw scores for MG‐ADL, MGC, and QMG and their changes expressed as reduction of each score are reported in Table 2.

**TABLE 2:** Raw scores for MG‐ADL, MGC, and QMG in 19 gMG patients treated with efgartigimod.

Clinical assessment	1st cycle, *n* = 19 patients		2nd cycle, *n* = 19 patients			3rd cycle, *n* = 10 patients			4th cycle, *n* = 5 patients			5th cycle, *n* = 4 patients		
	1st infusion	4th infusion	1st infusion	4th infusion	Follow‐up visit	1st infusion	4th infusion	Follow‐up visit	1st infusion	4th infusion	Follow‐up visit	1st infusion	4th infusion	Follow‐up visit
MG‐ADL raw score	8.2 (±3.1)	3.7 (±2.8)	3.4 (±2.8)	2.7 (±3.0)	3.9 (±3.5)	8.9 (±3.1)	5.0 (±4.1)	6.6 (±3.8)	10.4 (±4.0)	5.0 (±3.5)	7.3 (±2.8)	10.8 (±3.6)	4.3 (±2.4)	5.7 (±3.5)
MGC raw score	12.8 (±6.2)	5.5 (±5.2)	4.8 (±3.4)	4.4 (±4.8)	5.6 (±5.0)	14.3 (±4.4)	7.5 (±5.0)	8.3 (±6.1)	15.2 (±5.4)	5.8 (±5.3)	6.0 (±4.9)	13.3 (±8.4)	2.8 (±2.2)	5.3 (±4.7)
QMG raw score	11.6 (±4.8)	8.1 (±4.8)	9.3 (±4.9)	7.8 (±5.3)	8.9 (±5.7)	14.8 (±2.1)	11.3 (±4.1)	10.7 (±4.5)	15.0 (±2.8)	8.6 (±5.3)	11.8 (±4.9)	13.0 (±6.7)	8.5 (±5.7)	12.0 (±2.6)
Score changes														
MG‐ADL	–	−4.5 (±3.8)	−4.8 (±4.2)	−5.6 (±4.4)	−4.3 (±4.9)	–	−3.9 (±3.9)	−2.7 (±4.5)	–	−5.4 (±2.3)	−3.0 (±3.7)	–	−6.5 (±3.0)	−6.0 (±4.6)
MGC	–	−7.3 (±7.2)	−7.9 (±7.1)	−8.5 (±8.2)	−7.2 (±8.6)	–	−6.8 (±3.5)	−6.3 (±6.0)	–	−9.4 (±3.8)	−8.3 (±5.8)	–	−10.5 (±8.9)	−10.3 (±11.9)
QMG	–	−3.5 (±4.5)	−2.3 (±5.1)	−4.2 (±5.7)	−2.6 (±5.9)	–	−3.5 (±3.8)	−4.3 (±5.4)	–	−6.4 (±2.7)	−3.8 (±7.5)	–	−4.5 (±4.0)	−3.7 (±4.5)

*Note*: Values are expressed as mean ± SD.

Abbreviations: EFG, efgartigimod; gMG, generalized myasthenia gravis; MG‐ADL, Myasthenia Gravis Activity of Daily Living; MGC, Myasthenia Gravis Composite; QMG, Quantitative Myasthenia Gravis.

The Fixed period, administered to all 19 patients, was followed by clinical observation to evaluate the need for further treatment with EFG according to changes of the rating scales as reported. Ten of 19 (53%) patients received three cycles, five of 19 (26%) received four cycles, and four of 19 (21%) received five cycles along the clinical follow‐up of 14 months.

The time interval between treatment cycles during the Flexible period was variable among patients. The third cycle was administered after 11.3 ± 8.6 weeks, the fourth after 7.7 ± 2.2 weeks, and the fifth after 10.3 ± 2 weeks from the end of the previous treatment cycle (Figure 1b). As shown in Figure 1b, EFG retained its efficacy when readministered at each cycle during the Flexible period. Interestingly, worsening of the clinical scores occurred after a time interval that outlasted the known half‐life of circulating IgG in humans. The mean number of treatment cycles was 3.7 (±3.9) along the flexible period, and the annualized mean number was 3.1 (±3.3). It is noteworthy that nine patients did not need subsequent cycles; however, a longer follow‐up will be more informative on this issue.

**FIGURE 2:**
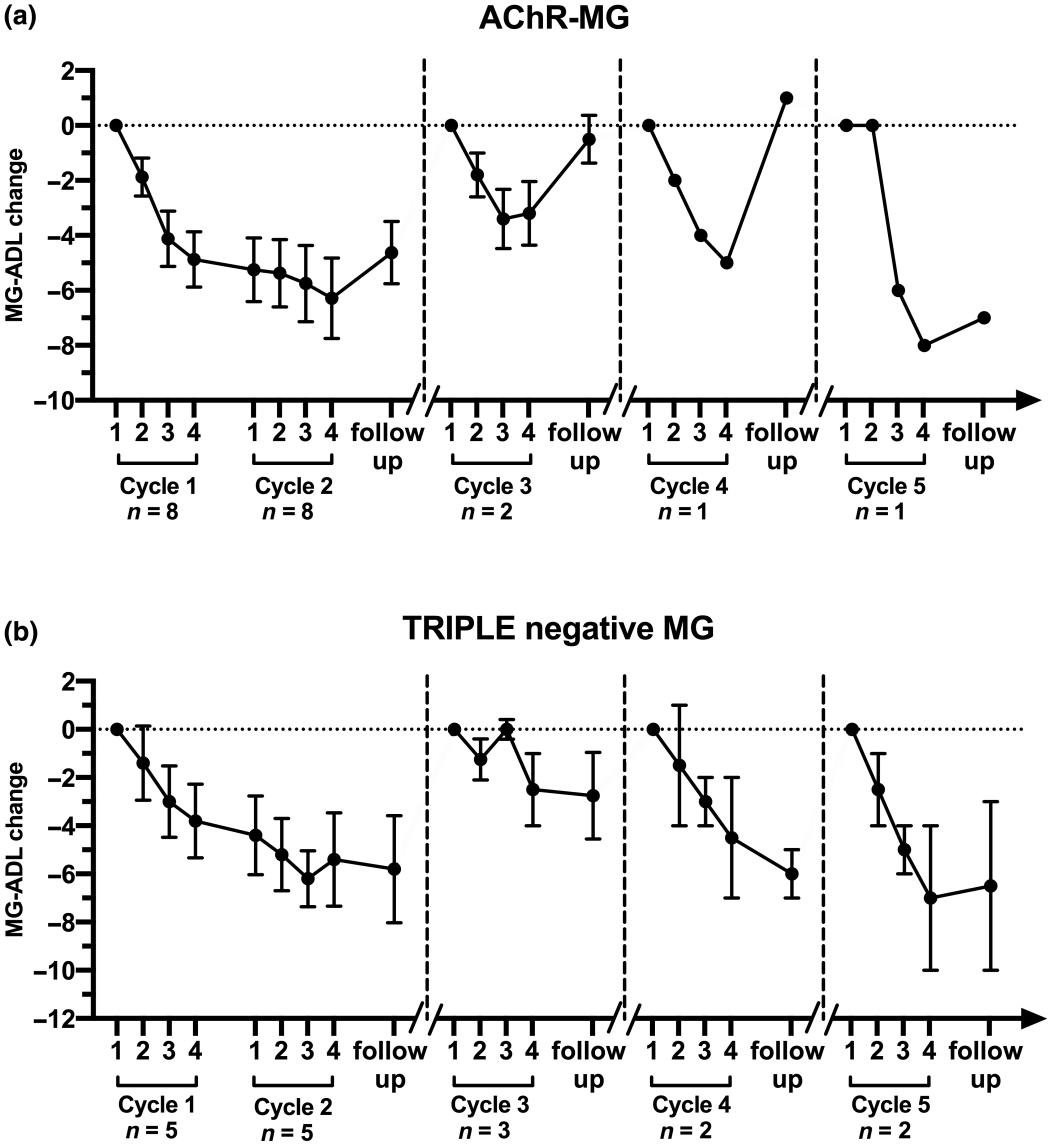
Myasthenia Gravis Activity of Daily Living (MG‐ADL) changes in acetylcholine receptor (AChR) antibody‐positive MG(a) and triple‐negative MG patients (b) treated with efgartigimod. MG‐ADL changes (expressed as reduction of the score values, mean ± SEM) were calculated comparing each time point to the score recorded at the first infusion of each cycle.

**FIGURE 3:**
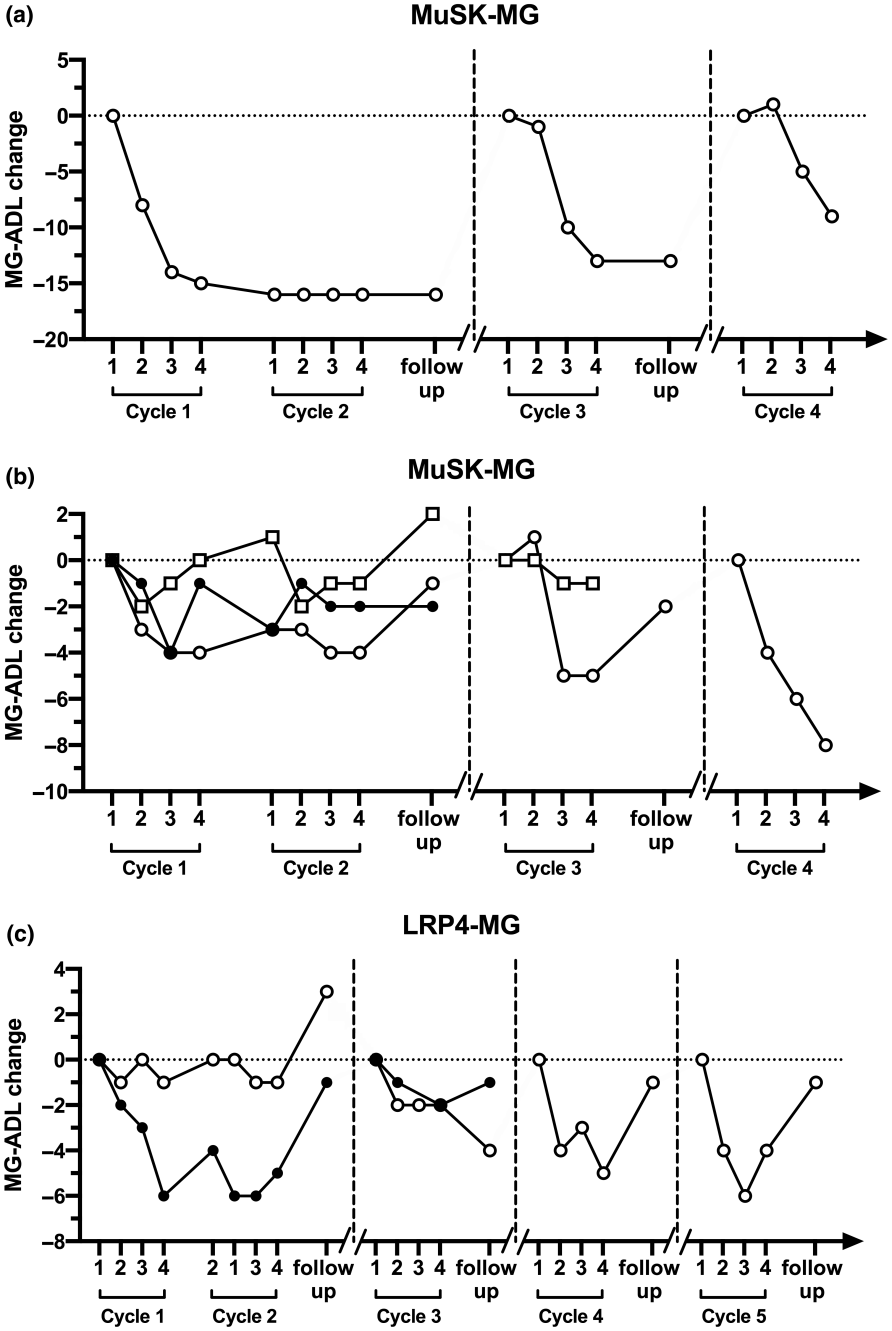
Myasthenia Gravis Activity of Daily Living (MG‐ADL) changes in muscle‐specific tyrosine kinase (MuSK; *n* = 4) and lipoprotein‐related protein 4 (LRP4; *n* = 2) generalized myasthenia gravis (MG) patients treated with efgartigimod (EFG). (a) MG‐ADL changes in an MuSK‐MG patient showing a rapid and considerable response to EFG. (b) MG‐ADL changes in the remaining three MuSK‐MG patients. (c) MG‐ADL changes in two LRP4‐MG patients. MG‐ADL changes were calculated comparing each time point to the score recorded at the first infusion of each cycle.

We also evaluated the clinical improvement in the gMG patients divided according to their autoantibody specificity. Interestingly, the course of clinical improvement induced by EFG was similar in anti‐AChR‐positive and triple‐negative gMG patients (Figure 2a,b). As far as MuSK MG patients are concerned, a repeated, striking response was observed in one MuSK‐MG patient, in whom the third and fourth treatment cycles were given 3 months apart (Figure 3a). A less prominent response was found in the remaining three MuSK‐MG patients (Figure 3b). We did observe some improvement in LRP4‐MG patients, but no definite conclusions could be drawn in two patients only.

**FIGURE 4:**
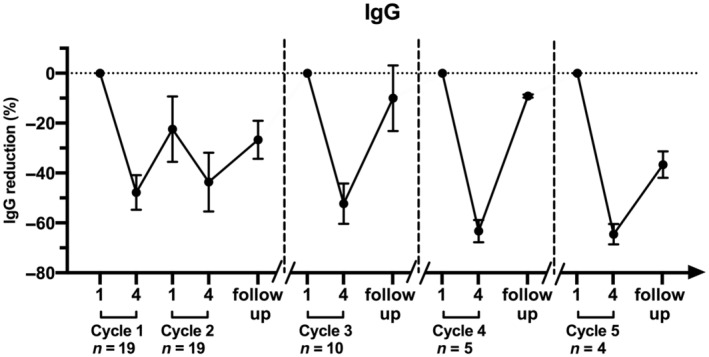
Total IgG changes (mean ± SEM) expressed as percentage reduction in blood samples collected at the first and fourth infusion of each efgartigimod cycle, and at each follow‐up visit 30 days after the end of each cycle.

EFG administration reduced both total IgG and specific autoantibodies. We measured total IgG and anti‐AChR antibodies in blood samples collected before the first and fourth infusion of the first and second cycle of the Fixed period and 30 days after the end of the Fixed treatment period. Total IgG, assayed in the whole group of 19 gMG patients before the fourth infusion of EFG, was reduced by 47.8% compared to basal values, and 30 days after the end the Fixed period was still reduced by 26.7%. The trend of total IgG reduction before and 30 days after the fourth infusion of the following cycles is reported in Figure 4. Fifty‐three percent reduction of anti‐AChR antibodies was found before the fourth infusion of the first cycle; 30 days after the end the Fixed period, anti‐AChR antibodies were reduced by 26%.

The impact of EFG on the course of the disease was dramatically evident when we considered the severity of MG in the year before enrollment in EAP, expressed as the need for hospitalization due to MG worsening, admissions to the intensive care unit, and need for immunomodulation with IVIG or Plex. In the year before starting EFG, eight of 19 (42%) patients were hospitalized, 15 of 19 (79%) needed variable treatment cycles with Plex or IVIG, and three of 19 (16%) needed admission to the intensive care unit. Interestingly, during treatment with EFG, none of the patients was admitted to the intensive care unit or hospitalized due to MG worsening, and only one patient needed immunomodulation with Plex or IVIG.

We assessed the postintervention status as reported by the MGFA [18] at the end of the clinical follow‐up of 14 months in our patients. Minimal manifestation status was recorded in three of 19 (16%), improved status (defined as MG‐ADL ≥ 2 or QMG ≥ 3) in 12/19 (63%), and unchanged/worsened status in four of 19 (21%).

**FIGURE 5:**
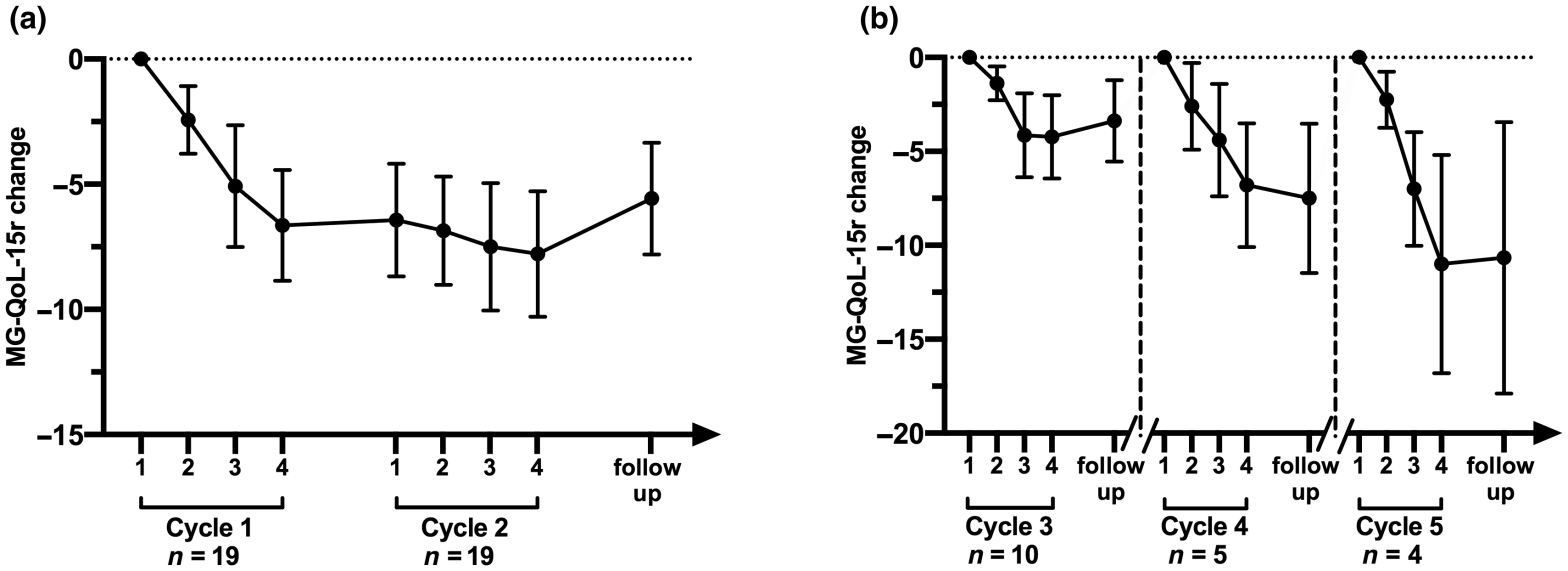
Revised 15‐item Myasthenia Gravis Quality of Life (MG‐QoL‐15r) changes in generalized myasthenia gravis patients treated with efgartigimod along the Fixed period (a) and Flexible period (b). MG‐QoL‐15r changes (expressed as reduction of the score values, mean ± SEM) were calculated comparing each time point to the score recorded at the first infusion of each cycle.

We also evaluated quality of life by means of the validated questionnaire MG‐QoL‐15r [24]. Data were available for 14 patients from baseline, and changes of the scores, reported in Figure 5, showed a positive trend, corresponding to the clinical scores.

As far as concomitant treatment is concerned, 15 of 19 patients (79%) were on prednisone treatment at the time of enrollment in the EAP program (Table 1). Treatment with EFG allowed nine of 15 (60%) patients to reduce the daily dose of prednisone; the mean reduction in the whole group of patients was 33 ± 34% over a period of time of 7.8 ± 3.4 months; the prednisone dose was increased in only one of 19 patients.

EFG was well tolerated. Only one serious adverse event occurred (complicated diverticulosis that needed surgical treatment), and it was considered unrelated to EFG treatment. The most frequently reported adverse events were headache (four patients, 21%), diarrhea (three patients, 15.8%), upper respiratory tract infections (five patients, 26.3%), and nausea (one patient, 5.2%). Two of the three patients who experienced diarrhea were not treated with anticholinesterase inhibitors. Interestingly, of patients who developed COVID‐19 infection (10/19, 53%), none had lung involvement or worsening of their myasthenic condition. All of them received anti‐COVID‐19 mRNA vaccine before entering the study.

## DISCUSSION

Real‐life management of MG has to cope with several issues, including comorbidities, side effects from long‐term immunosuppression, and treatment resistance, and should improve the patient's quality of life. Several decades of experience with traditional therapies, particularly corticosteroids, has highlighted the unmet need for effective, well‐tolerated, and targeted therapeutic options for MG. Moreover, the diagnosis of MG in the elderly has increased in recent years, making the treatment of the disease even more challenging [26].

The availability of several compounds acting specifically at different pathogenetic steps of the disease makes MG the optimal model for testing their efficacy, as occurred for anti‐FcRn inhibitors. EFG is the most investigated molecule so far among FcRn antagonists, and the first to be recently approved for clinical use in the USA, Japan, and Europe after successful randomized clinical trials [17].

We had the opportunity to study the efficacy of EFG outside controlled studies, according to the EAP program, in 19 generalized MG patients. We confirm the positive outcomes observed in clinical trials and further extend the observation of EFG efficacy over a longer clinical follow‐up in our patients. Meaningful clinical improvement was recorded early during the Fixed treatment period of the EAP, as observed in published randomized clinical trials [15, 16]. Afterward, EFG was given according to changes of clinical scores during the Flexible period and retained its efficacy, as shown in Figure 1. However, the need for readministration was variable, and the mean interval between treatment courses outlasted the half‐life of circulating IgGs, making EFG suitable for tailored treatment according to the individual clinical response. In this regard, it is worth noting that eight of 19 patients did not need retreatment after the Fixed period. Considering the time course of clinical improvement and the interval for retreatment observed during the clinical follow‐up, we speculate that in selected patients periodic EFG administration at fixed intervals is likely to prevent worsening or clinical fluctuations and should be further investigated. Results from the ADAPT+ study will probably provide some hint in this regard.

The trend of the clinical improvement observed in anti‐AChR and triple‐negative MG patients was similar; a positive response, particularly evident in one patient, was also found in MuSK‐MG.

The impact of EFG on the course of MG was even more impressive considering the severity of the disease during the year before enrollment in EAP, as the need for hospitalization and immunomodulation with IVIG or Plex was abolished except for one patient who needed immunomodulation during the Flexible period. The same positive trend occurred for corticosteroids, as 60% of patients on prednisone treatment were able to taper the dose up to 34% during the clinical follow‐up. It is also worth noting that treatment with EFG did not adversely affect the course of COVID‐19 infection in our vaccinated MG patients [27].

The positive experience with EFG along the clinical follow‐up allows several considerations regarding the management of gMG in the near future. Our experience provides evidence that EFG was safe and can be effective in the real‐world use. EFG represents a well‐tolerated therapeutic option that changed considerably the course of the disease in our patients, as it prevented the most severe forms of the disease, hospitalization, and respiratory insufficiency. The present data also expand the observation of improved quality of life in MG as reported in patients participating in the ADAPT study [28]. A longer follow‐up will be of particular interest, as it will provide further information regarding the actual impact on ongoing treatments, particularly corticosteroids, and on the overall quality of life of our patients.

A further positive aspect of FcRn inhibition is that EFG may be a viable alternative when IVIG are not available or Plex is not feasible due to contraindications or inadequate vascular access [29]. Moreover, considering the time course of improvement and the need for retreatment, EFG can be a promising approach for selected patients with comorbidities or major contraindications to traditional immunosuppression. In this regard, EFG has been investigated as add‐on therapy to date. However, the early administration of EFG in association with immunosuppressive drugs will be worth studying in MG to avoid as much as possible the use of corticosteroids or at least limit their use, being the major source of severe, long‐term side effects for MG patients [30].

Real‐world data on EFG in gMG are still very limited [31] Our experience has the weaknesses of being an open, uncontrolled study, on a small number of patients, but comes from real practice and included not only anti‐AchR and MuSK, but also LRP4 and triple‐negative MG patients, subgroups that were not considered by clinical trials but deserve more attention in future studies.

We therefore conclude that the era of targeted therapy for MG has finally begun and that, thanks to the specific compounds already in an advanced stage of investigation, the therapeutic scenario of MG is likely to change considerably in the next few years.

## AUTHOR CONTRIBUTIONS

**Carlo Antozzi:** Conceptualization; investigation; methodology; writing – original draft; data curation; supervision; writing – review and editing. **Rita Frangiamore:** Investigation; data curation. **Elena Rinaldi:** Formal analysis; data curation. **Fiammetta Vanoli:** Investigation. **Francesca Andreetta:** Investigation. **Emilio Ciusani:** Investigation. **Silvia Bonanno:** Investigation. **Lorenzo Maggi:** Investigation. **Annamaria Gallone:** Investigation. **Anna Colasuonno:** Data curation. **Irene Tramacere:** Methodology; formal analysis. **Marta Cheli:** Investigation. **Alessandro Pinna:** Supervision. **Renato Mantegazza:** Conceptualization; supervision; methodology.

## FUNDING INFORMATION

R.M. is the recipient of an MoH annual grant and ERAPERMED2022‐258, GA 779282. argenx provided EFG for treatment of MG patients included in the study, according to the Expanded Early Access Program for Efgartigimod.

## CONFLICT OF INTEREST STATEMENT

F.V. has received funding for consulting and speaking from Alexion Pharmaceuticals and argenx. L.M. has received funding for travel, meeting attendance, and advisory board participation from Sanofi Genzyme, Roche, Biogen, Amicus Therapeutics, Alexion, Janssen, Lupin, and argenx. S.B. has received funding for travel, meeting attendance, and advisory board participation from Sanofi Genzyme, Biogen, Alexion, and Roche. R.M. has received funding for travel, meeting attendance and advisory board participation from Alexion, argenx, BioMarin, Catalyst, Sanofi Genzyme, Regeneron, and UCB. C.A. has received funding for travel, meeting attendance, and advisory board participation from Alexion, Momenta, Sanofi, argenx, and UCB. A.P. is an employee of argenx. None of the other authors has any conflict of interest to disclose.

## Data Availability

The data that support the findings of this study are available from the corresponding author upon reasonable request.
